# Life paths of familial amiloidotic polyneuropathy patients: a descriptive study

**DOI:** 10.1186/1750-1172-10-S1-P56

**Published:** 2015-11-02

**Authors:** Alice Lopes, Alexandra Sousa, Isabel Fonseca, Margarida Branco, Carla Rodrigues, Paula Freitas, Teresa Coelho

**Affiliations:** 1 Centro Hospitalar do Porto, Corino de Andrade Unit, 4099-001, Porto, Portugal; 2 Universidade do Porto, Instituto de Ciências Biomédicas Abel Salazar, 4050-313, Porto, Portugal

## Background

Very few studies describe the relations between biographic events, socio-familiar characteristics, and disease during the lifetime of familial amyloid polyneuropathy (FAP) patients.

## Methods

A common social demographic questionnaire and a questionnaire about family history/personal disease and biographic events were applied to 211 subjects (in 110, the disease had already begun, 82 were asymptomatic carriers, and 19 had no established diagnosis), attending external consultation at Corino de Andrade Unit in Centro Hospitalar do Porto.

A descriptive analysis and frequencies were obtained.

## Results

84 of all subjects were men and 127 were female. Mean ages: carriers 33.9 ± 9.8yr, patients 37.8 ± 8.1, and for subjects that had no established diagnosis 40.9 ± 14.0 yr. Most subjects were married or lived with a partner (67.1%); 61.5% had children (mean, 4 children).

Most subjects (96.3%) had contacted the disease before having their diagnosis, most of them through parents (35.7%) but also through uncles, grandparents, other family members; the affected parent was the mother in 53.8% of the cases and the father in 43.3% of the patients (the remaining did not know who the affected parent was; one patient inherited the disease from both parents); 71.8% were deceased. Most living parents had symptoms (74.4%).

Age at time of the affected parent's death: most of the subjects were older than 25 yr (43%); the remaining were under 10 yr (9.9%); between 10 and 14 yr (15.5%); and between 15 and 24 yr.

Age at time of the parent's disease onset: most of the subjects were under 10 yr (30.5%); the remaining were between 10 and 14 yr (14.4%); between 15 and 24 yr (27.0%); and 17.2% were older than 25 yr.

When asked about whether and how their parent's disease had brought changes into their lives, 37.2% of the subjects said yes, namely through residence, psychological and familial questions.

Most subjects (53.3%) had been their parent´s caregivers.

About 7.5 % of all patients had been raised through childhood and youth by others, not by their parents.

Some subjects (8.4%) refused to acknowledge their own genetic test's result for more than 1 year.

## Discussion and conclusions

Parent´s death and the presence of an early-onset disease is a constant in FAP patients´ lives and this may be an important distress factor, eventually making them more vulnerable to psychological distress and psychiatric disease.

During childhood, youth, and as young adults, a great number of these patients were obliged to become caregivers and this implied a change of roles in the family.

These results point to a very important psychosocial charge that FAP imposes to patients throughout their lives, since childhood and youth.

